# *In silico* identification and experimental validation of shared genes and exploration of molecular links between type 2 and non-type 2 asthma

**DOI:** 10.3389/fmed.2026.1798063

**Published:** 2026-05-08

**Authors:** Zaixing Jia, Bin Liu, Jing Cao, Siqin Han, Shujie Hou, Zixiao Chen, Jialun Chen, Zhenwei Liu, Weihua Chen, Jingwen Li, Rongqin Li, Xixin Yan

**Affiliations:** 1 The First Department of Pulmonary and Critical Care Medicine, The Second Hospital of Hebei Medical University, Shijiazhuang, Hebei, China; 2 Hebei Key Laboratory of Respiratory Critical Care Medicine, Hebei Institute of Respiratory Diseases, Shijiazhuang, Hebei, China; 3 Central Laboratory, The Second Hospital of Hebei Medical University, Shijiazhuang, Hebei, China

**Keywords:** asthma, bioinformatics, common genes, non-T2, T2

## Abstract

**Objective:**

Asthma, a complex disease, is categorized into type 2 (T2) and non-type 2 (non-T2) molecular endotypes. Growing evidence supports the overlap between endotypes and highlights a complex interplay among multiple inflammatory cell types. Combined inflammatory disease is particularly challenging to treat. Building on the background of overlapping asthma endotypes, the goal of this research was to explore common gene signatures and the underlying molecular mechanisms across different asthma subtypes.

**Methods:**

Transcriptomics data of bronchial biopsy samples were sourced from the Gene Expression Omnibus (GEO) and intersected to identify common genes in T2 and non-T2 Asthma. Gene ontology, pathway enrichment, protein–protein interaction, immune infiltration, and single-cell analysis were used to explore the roles of these genes. Finally, common genes in T2 and non-T2 asthma mice models were verified by RT-qPCR.

**Results:**

Five upregulated common genes (MS4A2, TPSAB1, FCER1A, TFF3, TSPAN13) were identified. These genes showed good diagnostic accuracy for both T2 and non-T2 asthma. Gene Set Enrichment Analysis (GSEA) analyses revealed involvement of these genes in the immune and inflammatory response. Moreover, we found that both T2 and non-T2 asthma exhibit similar patterns of immune cell infiltration, such as mast cells, suggesting that mast cell activation can span different inflammatory phenotypes. Consistently, qRT-PCR analysis confirmed that the five genes were upregulated in both T2 and non-T2 asthma, aligning with the bioinformatic predictions.

**Conclusion:**

This study revealed common genes and similar immune cell infiltration microenvironments in both T2 and non-T2 asthma. These findings may provide new insights for future studies on molecular mechanisms.

## 1 Introduction

Asthma is a complex, chronic disorder characterized by airway inflammation and obstruction. About 350 million disorder worldwide currently suffer from asthma (1, 2). As a highly heterogeneous disease, it is caused by the interaction between allergens and other external irritants, as well as the host immune response (3). While most asthmatic patients can effectively relieve symptoms with corticosteroids and bronchodilators, a subset of approximately 5–10% suffer from uncontrolled asthma (4).

In recent years, with the advancement of asthma pathobiology and molecular biology, an increasing number of scholars have begun to explore the T2- Non-T2 Interplay in the Complex Immune Landscape of Severe Asthma. Allergic and eosinophilic asthma are subpopulations driven by T2 inflammation with overlapping features (5). Blood eosinophils, Immunoglobulin E (IgE), and fractional exhaled nitric oxide (FeNO) are most commonly used biomarkers of T2 asthma (6). A large body of evidence suggests that immunity and inflammation are noteworthy factors contributing to the development of T2 asthma (7, 8). Non-T2 asthma is characterized by being nonallergic, noneosinophilic, and “T2-low (L)” asthma (9). It is typically characterized by steroid resistance and the absence of T2 asthma biomarkers, making diagnosis difficult (10). Non-T2 asthma encompasses various subtypes, including neutrophilic and paucigranulocytic asthma (11). Non-T2 airway inflammation has been linked to several biological mechanisms, including inflammasome activation and increased activation of Th1 and/or Th17 cells (7). Some studies have suggested that the simultaneous presence of T1 and T2 immune responses may be responsible for the poor responsiveness to corticosteroids of T2 asthma (12, 13). Analysis of airway brushings by RNA sequencing from individuals with mild asthma revealed elevated T2 and T1 signals compared to healthy controls (14). Another extensive cohort study further supports this notion, demonstrating that T1-High/T2-High (H) severe asthma patients have a higher Exhaled Nitric Oxide (FeNO) compared to T1-L/T2-H individuals, and corticosteroid-associated improvements forced expiratory volume at 1 s (FEV1) did not occur (15). Notably, a report examining biomarkers of clinical response to mepolizumab and omalizumab showed that patients with inflammation in mixed T1/T2 pathways are less likely to respond to these therapies (16). Although existing evidence points to an association between T2 and non-T2 asthma, further studies are required to clarify the precise molecular mechanisms and establish causal relationships. In this regard, immune and inflammatory pathways may serve as key links connecting these two asthma endotypes.

Collectively, these findings highlight a significant interaction between T1 and T2 inflammatory pathways in asthma. While associations between T1 and T2 responses have been frequently reported, the specific molecular pathways bridging these immune phenotypes remain poorly understood. This study aims to identify key shared genes between T2 and non-T2 asthma. Through correlation analyses and protein–protein interaction (PPI) network construction, we have identified cross-talk genes at the interface of T2 and non-T2 asthma and investigated the interactive mechanisms between these subtypes. Furthermore, we validated these shared genes in an experimental asthma model, offering new perspectives on asthma management and therapeutic approaches.

## 2 Materials and methods

### 2.1 Data collection

The GSE143303, and GSE147878 datasets were obtained from the publicly accessible Gene Expression Omnibus (GEO) database (available at https://www.ncbi.nlm.nih.gov/geo/). Among them, we have designated GSE143303 as the test set, and GSE147878 as the validation sets. GSE143303 dataset: This collection consists of 60 bronchial biopsy specimens. These include 13 samples from healthy individuals, 9 from patients diagnosed with neutrophilic asthma, and 38 from cases of non-neutrophilic asthma. The non-neutrophilic group is further subdivided into 22 eosinophilic asthma and 16 paucigranulocytic asthma samples. GSE147878 dataset: This dataset contains 73 bronchial biopsy samples. The cohort is composed of 13 healthy controls, 18 individuals with mild to moderate asthma, and 42 patients with severe asthma.

### 2.2 Identification of DEGs between T2 and non-T2 endotypes

In the GSE143303 dataset, patients with eosinophilic asthma were categorized as T2 endotypes, whereas those with neutrophilic asthma and paucigranulocytic asthma were designated as non-T2 endotypes. Following batch correction, the limma software package (version 3.52.4) was employed to analyze the corrected T2 and non-T2 endotypes and identify differentially expressed genes (DEGs), applying thresholds of |logFC| > 0.5 and an adjusted *p*-value < 0.05. The expression patterns of the DEGs were visualized using the ggpubr, ggthemes, and pheatmap packages in R.

### 2.3 Pathway enrichment and gene ontology analyses

For this investigation, the DEGs associated with T2 and non-T2 endotypes were submitted to the Metascape database for Gene Ontology (GO) functional enrichment analysis and Kyoto Encyclopedia of Genes and Genomes (KEGG) pathway analysis. The analysis parameters were established with a *p*-value <0.05, a minimum overlap of 3, and a minimum enrichment score of 1.5. These criteria were selected to ensure both statistical significance and biological relevance of the enrichment outcomes.

### 2.4 Determine hub genes using machine learning

Three machine learning methods (Least AbsoluteShrinkage and Selection Operator (Lasso) regression, Random Forest (RF), and Support Vector Machine-Recursive Feature Elimination (SVM-RFE)) were used to screen for common genes. SVM can be used for classification and regression (17), RF algorithm was implemented using the randomForest package, and LASSO is based on the R package “glmnet” (18). We utilize the LASSO algorithm to identify potential shared genes. The SVM-RFE technique employs recursion to rank features and prevent overfitting. The random forest algorithm is used to rank gene importance. Use the R package “Venn” to screen key genes by intersecting gene sets obtained from ≥2 methods.

### 2.5 Identification of the common genes of T2 and non-T2 endotypes

The limma R package was utilized to process and analyze the GSE147878 dataset. The intersection of differentially expressed genes was verified using the GSE 147878 datasets for their expression in bronchial biopsy tissues and bronchial epithelial cells of asthma patients.

### 2.6 Protein–protein interaction networks

To investigate the common genes and other genes with related functions, protein–protein interaction (PPI) networks were constructed using GeneMANIA.^1^ GeneMANIA is a robust tool that integrates data from various sources to analyze gene–gene interactions. All genes within the PPI network were selected for further analysis.

### 2.7 Analysis of gene-disease interaction networks

The DISGENET database facilitates the exploration of similarities among diseases or between diseases and traits by analyzing shared genes and variants, covering the full range of human diseases along with normal and abnormal characteristics. A gene-disease network was constructed to investigate asthma-related diseases using the NetworkAnalyst platform.

### 2.8 Gene set enrichment analysis

Spearman correlation analysis was conducted between hub genes and the remaining genes across samples with the R package “psych,” producing correlation coefficients. Genes were subsequently ranked based on these coefficients, resulting in gene lists linked to each biomarker. GSEA was performed using the sorted outcomes and the R package “ClusterProfiler.” The five most significant signaling pathways were visualized with the gseaplot2 function (*p* < 0.05 and |Normalized Enrichment Score (NES)| > 1).

### 2.9 Receiver operating characteristic curve analysis

The pROC software package (version 4.1.3) in R was employed to generate ROC curves for assessing the predictive values of hub genes. The ROC curve was established using the external dataset GSE147878. The area under the curve (AUC) and 95% confidence intervals (CI) were computed, with AUC values >0.8, 0.6–0.8, and 0.5–0.6 indicating high, moderate, and low differentiation levels, respectively.

### 2.10 Immune infiltration analysis

To examine the infiltration status of various immune cells in T2 and non-T2 groups, the CIBERSORT algorithm was applied to quantify the levels of 22 immune cell types. This analysis aimed to identify the most relevant immune cell. Pearson correlation coefficients were calculated between the abundance of different immune cell types and the expression of hub genes identified in the study, with visualization achieved using the Vioplot and heatmap R packages.

### 2.11 Single-cell RNA sequencing dataset processing

Initially, the scRNA-seq data (GSE164015) were processed using the “Seurat” software package (version 5.1.0). The filtering criteria were as follows: nFeature_RNA > 300, nFeature_RNA < 5,000, percent.mt < 20, and nCount_RNA < 50,000. Clusters were then annotated using the dimensionality reduction technique of Uniform Manifold Approximation and Projection (UMAP). These clusters were assigned to distinct cell subpopulations with significant relevance to the disease. Finally, the FindAllMarkers feature combined with the Wilcoxon rank-sum test was utilized to identify marker genes for each cluster within the single-cell gene expression profile.

### 2.12 Cell culture and treatment conditions

Human bronchial epithelial (HBE) cells, obtained from the American Type Culture Collection (ATCC), were maintained in Dulbecco’s Modified Eagle Medium (DMEM; Gibco, United States) supplemented with 10% fetal bovine serum (FBS; Gibco, United States) and 1% penicillin/streptomycin (Solarbio, China). The cells were incubated at 37 °C in a humidified environment with 5% CO₂. After seeding in complete medium for 24 h, cells were exposed separately to house dust mite extract (HDM; 30 μg/mL, Dermatophagoides pteronyssinus, Greer Laboratories, United States), fine particulate matter (PM2.5; 50 μg/mL), or toluene diisocyanate (TDI; 100 μM, Sigma-Aldrich, United States). Following 24 h of treatment, cells were collected for quantitative reverse transcription polymerase chain reaction (qRT-PCR) analysis.

### 2.13 Development of an asthma animal model

Male C57BL/6 mice (WT, 6 weeks old, weighing 20–25 g) were sourced from Beijing Weitong Lihua Experimental Animal Technology Co., Ltd. All animals were acclimatized for 1 week under standard housing conditions with ad libitum access to food and water before the start of experiments. The study protocol was reviewed and approved by the Animal Ethics and Experiments Committee of the Second Hospital of Hebei Medical University (Approval No. 2024-AE287).

For asthma induction, mice were randomly assigned to four experimental groups (A–D), each consisting of six animals: a control group, an eosinophilic asthma (T2-high) group, a neutrophilic asthma (non-T2) group, and a mixed granulocytic asthma (non-T2) group.

A (control group): Male C57BL/6 mice of WT were kept in individually ventilated cages and exposed to filtered fresh air.

B (eosinophilic asthma group, HDM): Each mouse in the eosinophilic asthma group was intranasally administered a dose of 20 μg/40 μL HDM (D. pteronyssinus, Greerlabs, United States) in the sensitization stage (day 0 and day 14) and in the challenge stage (day 21, 23, 25, 27, and 29) (19).

C (neutrophilic asthma group, PM2.5): PM2.5 was suspended at a concentration of 1 mg/mL in sterile saline with 0.02% Tween-80 p.m. particle solution. Mice were exposed to 50 μL of PM2.5 for four h (days 1, 3, and 5) via oropharyngeal aspiration (OA) (20). Association between particulate matter containing EPFRs and neutrophilic asthma through AhR and Th1.

D (mixed granulocytic asthma group, TDI): Briefly, on days 1 and 8, mice were immunized intraperitoneally with 0.3% TDI on the dorsum of both ears (20 μL per ear). At days 15, 18, and 21, the mice were challenged for 3 hours each time with 3% TDI oropharyngeal aspiration. TDI was dissolved in a mixture of 3 volumes of olive oil and two volumes of acetone for sensitization and in a mixture of 4 volumes of olive oil and 1 volume of acetone for challenge (21).

### 2.14 BALF cell counts and histological examination of lungs in mice

Corresponding lung tissue sections were prepared through a series of steps including dehydration, embedding, and sectioning. Following the manufacturer’s guidelines, lung sections were stained with hematoxylin and eosin (H&E) and periodic acid-Schiff (PAS). Semiquantitative scoring systems, as previously described (19), were applied to assess the degree of lung inflammation and mucus secretion based on H&E and PAS staining results.

Bronchoalveolar lavage fluid (BALF) cell pellets were stained with Trypan Blue and counted by three independent blinded investigators. A total of 200 cells were counted to determine the percentage of eosinophils and neutrophils in the BALF fluid.

### 2.15 Quantitative reverse transcription polymerase chain reaction

Total RNA was isolated from cells and lung tissues using TRIzol reagent (Invitrogen, United States). Reverse transcription of extracted RNA into complementary DNA (cDNA) was performed with the Evo M-MLV RT Reaction Mix Kit (Accurate Biotechnology, China) in accordance with the manufacturer’s instructions. Subsequently, real-time quantitative polymerase chain reaction (qPCR) was carried out using the SYBR Green Premix Pro Taq HS qPCR Tracking Kit (Accurate Biotechnology, China) on a Bio-Rad CFX PCR System (Bio-Rad, United States). GAPDH and *β*-ACTIN served as internal reference genes for normalization. Relative gene expression levels were calculated via the 2−ΔΔCT method. Primer sequences used in the qRT-PCR assays are provided in Supplementary Table S1.

### 2.16 Statistical analysis

Student’s *t*-test, Wald Chi-Squared test, and Wilcoxon rank sum test were applied as specified. Correlation analysis was completed with the Spearman method. The statistical analyses of qRT-PCR were performed using GraphPad Prism (San Diego, CA, United States). Results were presented as mean ± standard deviation (SD). Differences between groups were analyzed using one-way ANOVA. The Student–Newman–Keuls (SNK) method was employed to perform pairwise comparisons with homogeneity of variance. All experiments were replicated at least three times. A value of *p* < 0.05 was considered statistically significant and denoted with 1, 2, 3, or 4 asterisks when <0.05, 0.01, or 0.001, respectively.

## 3 Results

### 3.1 Identification and functional enrichment of differentially expressed genes

**Figure 1:**
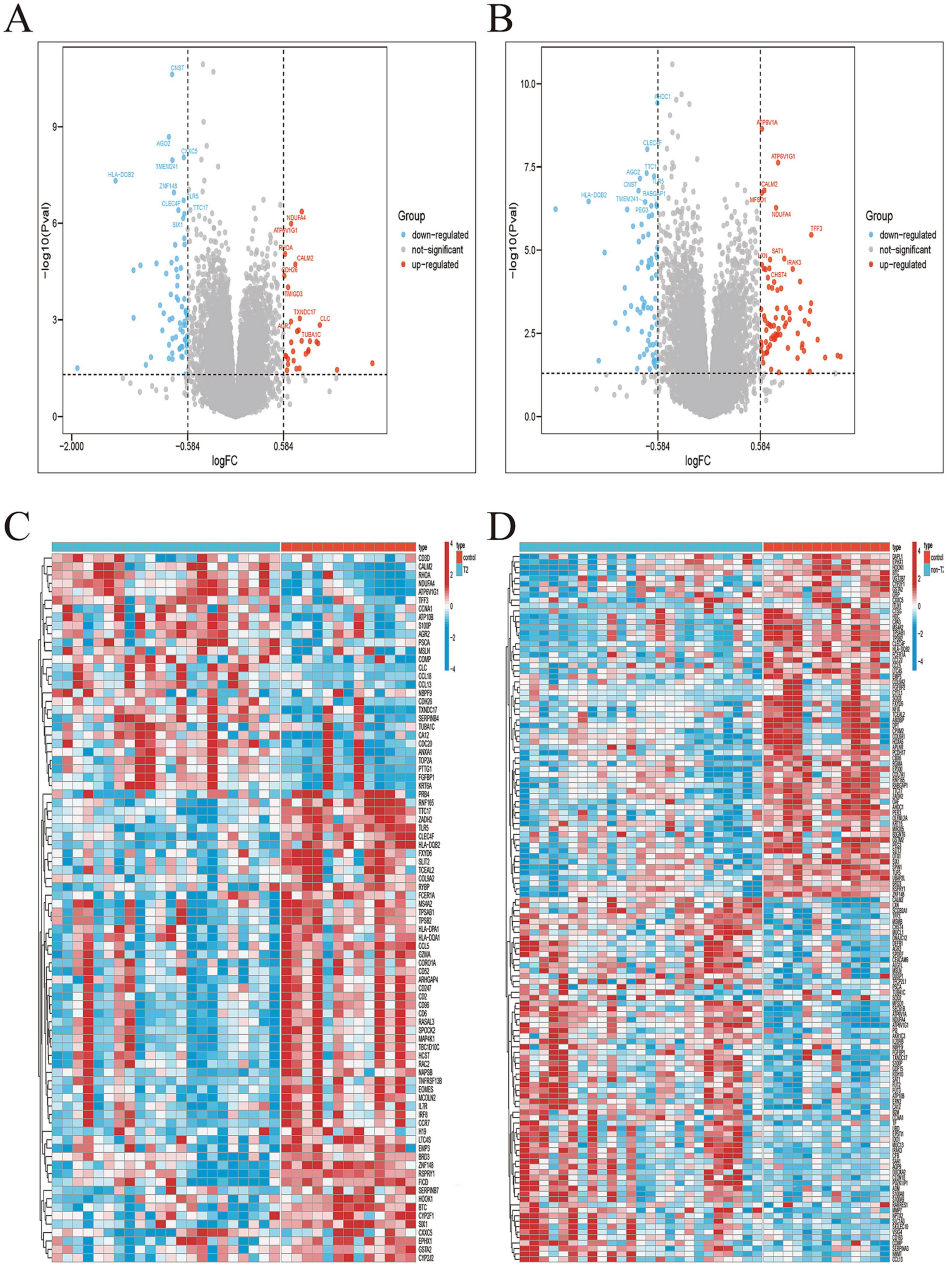
Identification of differentially expressed genes. **(A)** The volcano plots of T2 DEGs; **(B)** the heatmap of T2 up-regulated and down-regulated genes. **(C)** The volcano plots of non-T2 DEGs; **(D)** the heatmap of non-T2 up-regulated and down-regulated genes.

Analysis of differentially expressed genes (DEGs) between T2 and non-T2 asthma endotypes revealed distinct transcriptional profiles. In the T2 endotype, 30 genes were upregulated and 66 were downregulated, while the non-T2 endotype exhibited 74 upregulated and 70 downregulated genes. The expression patterns of these DEGs were visualized using volcano plots and heatmaps (Figures 1A–D).

**Figure 2:**
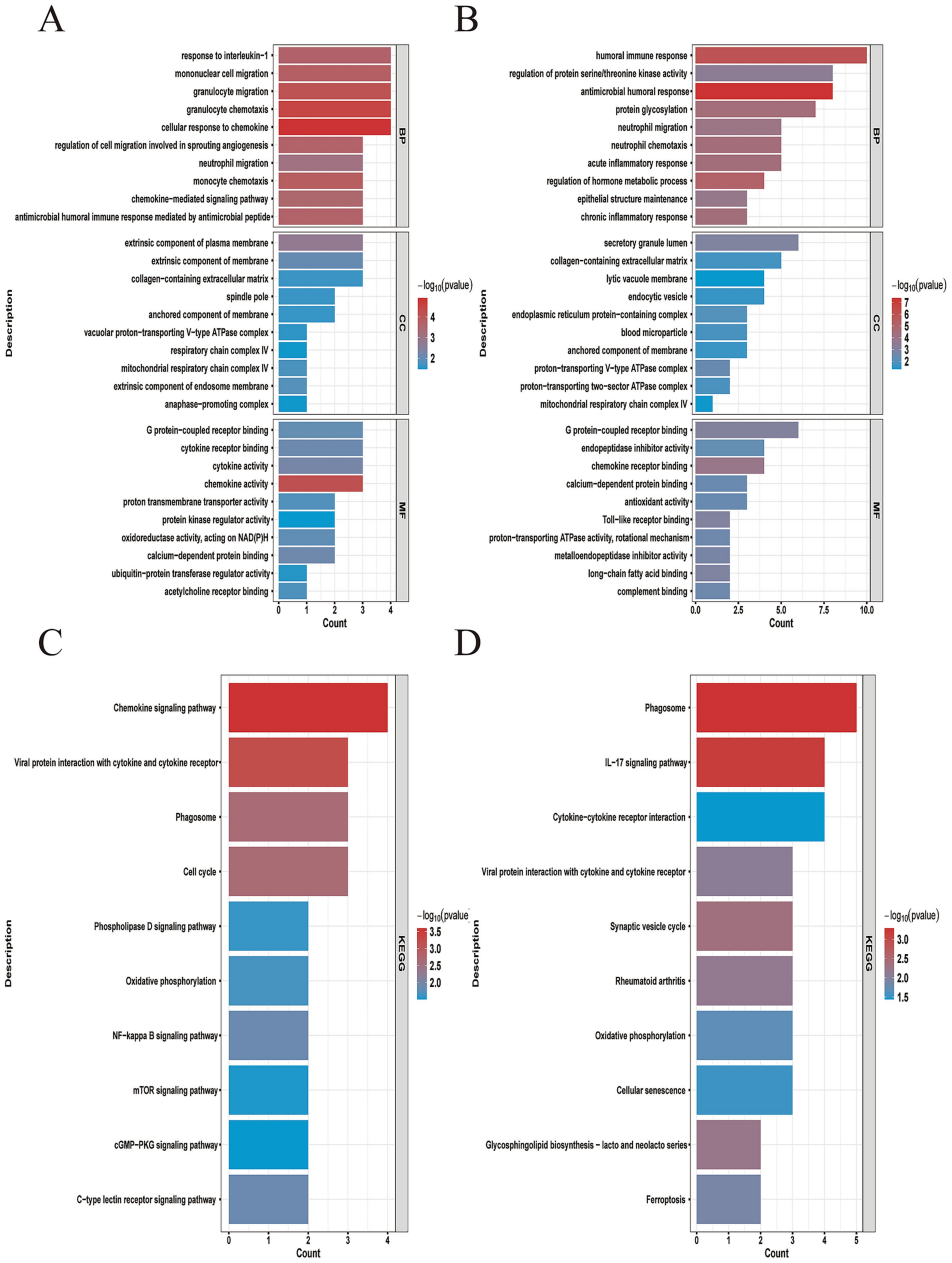
Gene ontology (GO) and Kyoto encyclopedia of genes and genomes (KEGG) enrichment annotations of the DEGs. **(A)** The results of GO enrichment categories in T2 included biological process (BP), cellular component (CC), and molecular function (MF). **(C)** The results of KEGG pathway enrichment analyses in the T2. **(B)** The results of GO enrichment categories in non-T2 included biological process (BP), cellular component (CC), and molecular function (MF). **(D)** The results of KEGG pathway enrichment analyses in the non-T2.

Gene Ontology (GO) enrichment and KEGG pathway analyses indicated that DEGs in both T2 and non-T2 asthma were significantly associated with biological processes and pathways such as “humoral immune response,” “IL-17 signaling pathway,” “chemokine signaling pathway,” and “acute inflammatory response” (Figures 2A–D). Collectively, these findings underscore the central involvement of immune and inflammatory mechanisms in the shared pathogenic pathways of T2 and non-T2 asthma.

### 3.2 Determine common genes using machine learning

**Figure 3:**
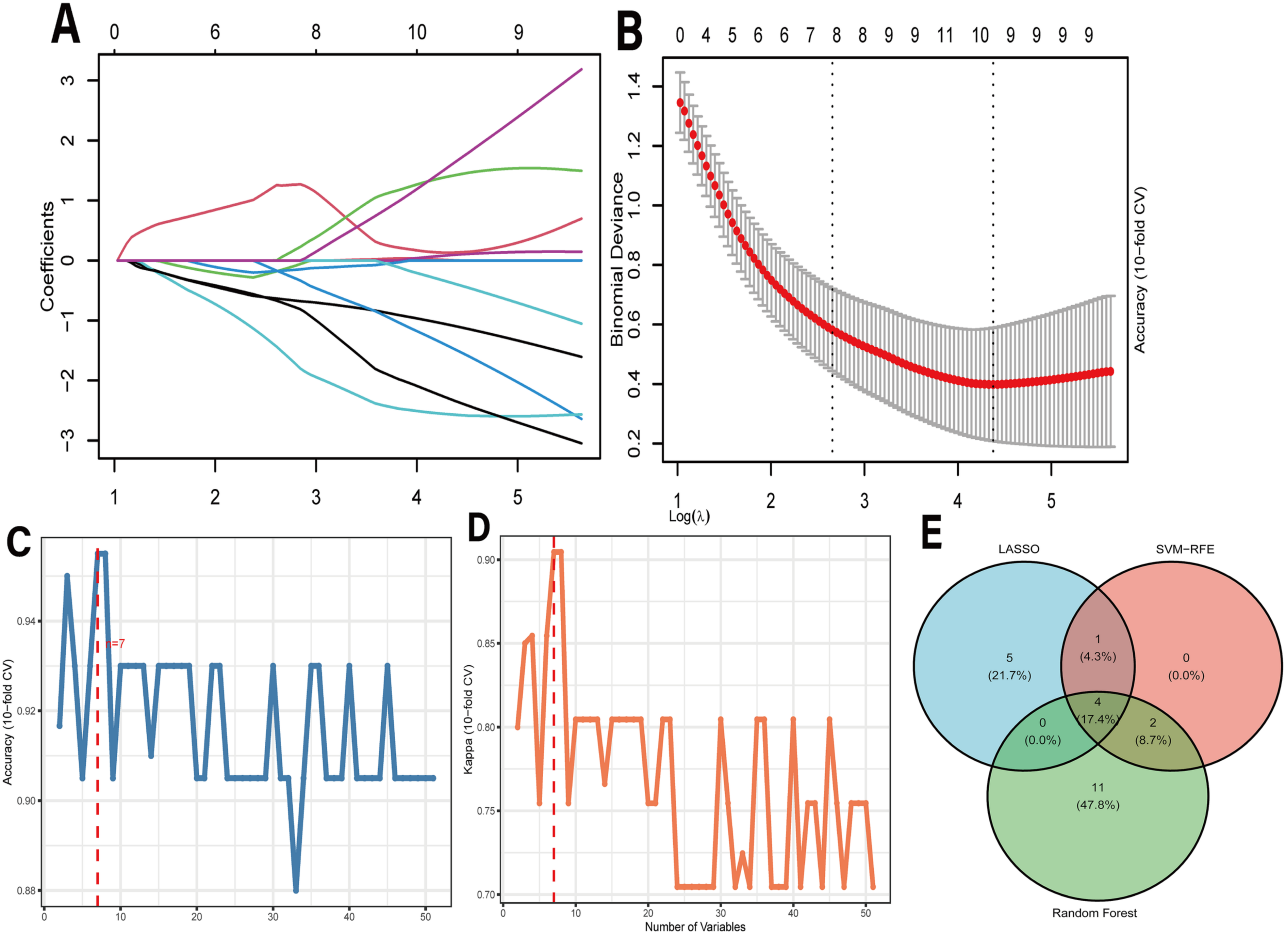
Machine learning for screening common genes. **(A,B)** Lasso algorithm; **(C,D)** SVM-RFE algorithm; **(E)** The obtained signature genes from the intersection of the RF, lasso, and SVM-RFE methods.

The LASSO regression was cross-validated using a 10-fold cross-validation, and 10 non-zero-coefficient genes were selected using lambda. Min. SVM-RFE achieved its peak accuracy (approximately 94%) with 7 variables in cross-validation, thereby identifying 7 optimal feature genes. Random Forest ranked features by Mean Decrease Gini, and 17 genes exceeded the mean threshold. Based on the multiple-algorithm voting consensus strategy, 6 hub genes were finally identified (CNST, TPSAB1, TFF3, MS4A2, TSPAN13, and FCER1A) (Figure 3).

### 3.3 Protein–protein interaction network analysis and external dataset validation of the expression

**Figure 4:**
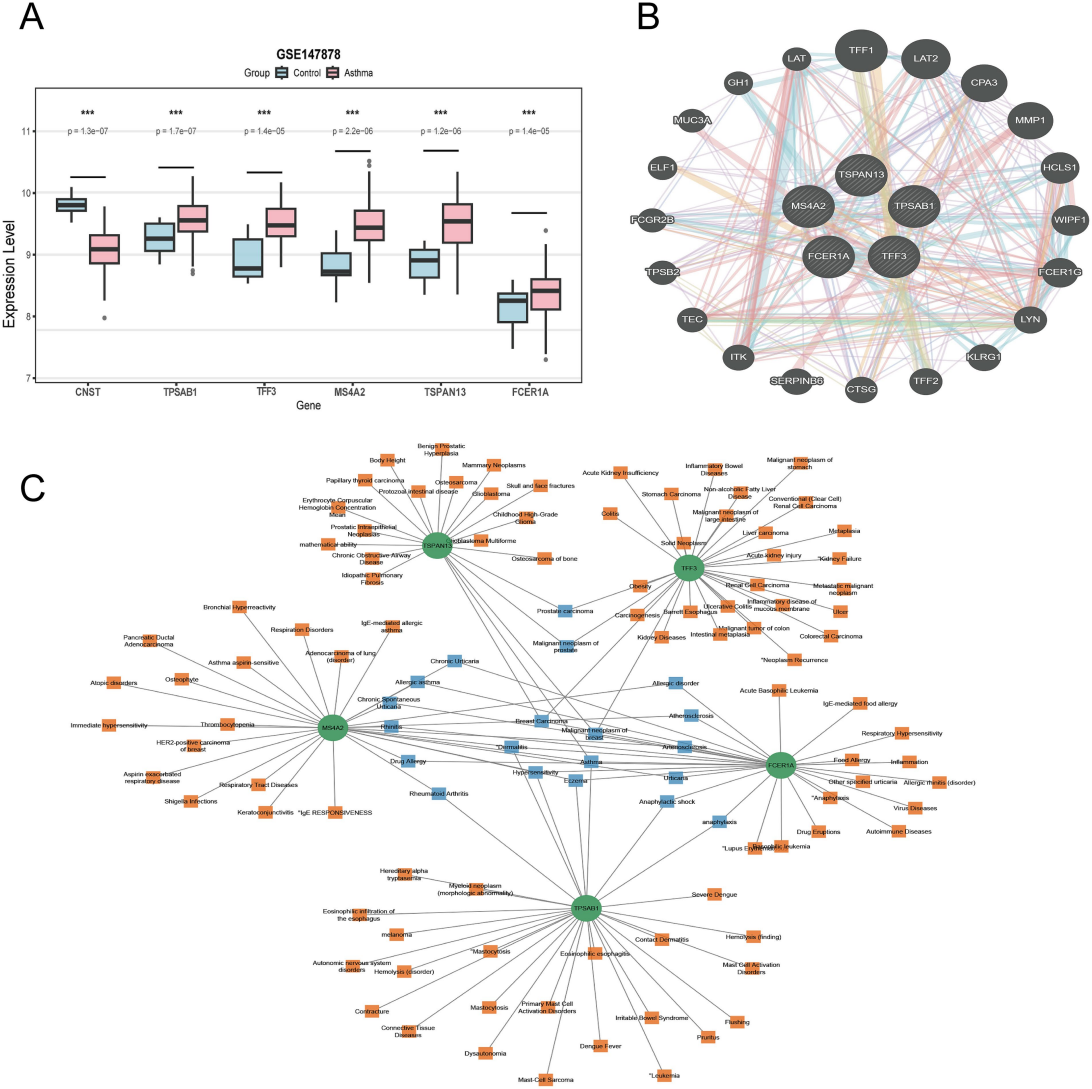
Identification of common genes related to diseases, and PPI network. **(A)** Expression of common genes in validation dataset GSE147878. **(B)** Interaction networks of key genes and proteins. **(C)** Gene-disease interaction network analysis. Dots represent hub genes; square dots represent diseases related to hub genes.

The six common genes was verified in the external datasets GSE147878 (Figure 4A). In the external dataset GSE147878, the expression levels of TPSAB1, TFF3, MS4A2, TSPAN13, and FCER1A were higher in asthma samples than in normal control samples. To investigate functional relationships among these overlapping genes, a protein–protein interaction (PPI) network was constructed using GeneMANIA. From this network, five hub genes were extracted (Figure 4B). The PPI network analysis underscores the functional connectivity and potential collaborative roles of these genes in asthma pathogenesis (Figure 4C).

### 3.4 Gene set enrichment analysis of key genes

**Figure 5:**
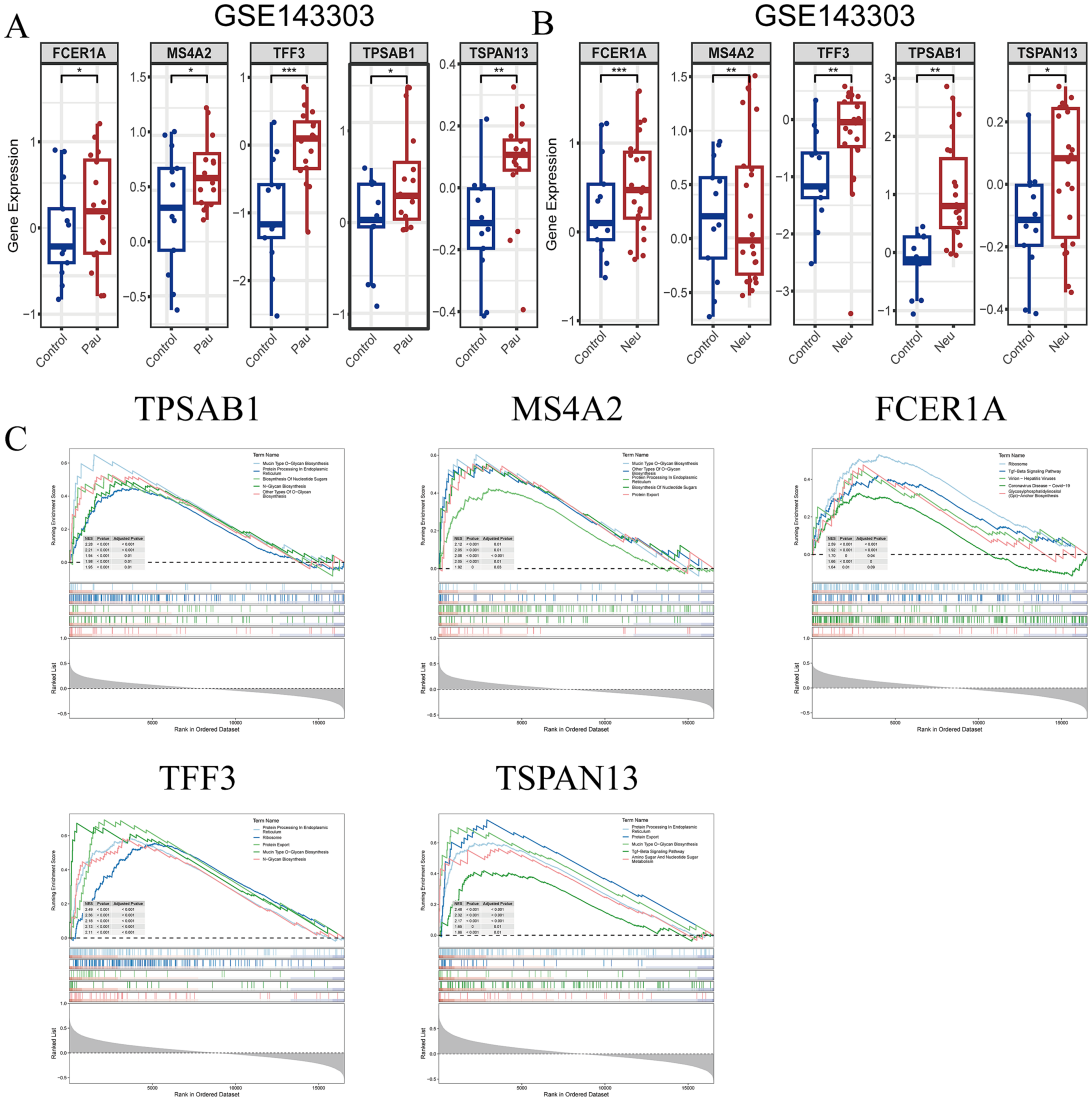
Differences in the expression of common genes in neutrophilic asthma and paucigranulocytic asthma from dataset GSE143303, and enrichment analysis. **(A,B)** Expression difference of common genes between neutrophilic asthma and paucigranulocytic asthma from dataset GSE143303. **(C)** GSEA enrichment analysis of the common genes.

In the test set GSE143303, the expression of TPSAB1, TFF3, MS4A2, TSPAN13, and FCER1A showed significant differences between healthy individuals and patients with paucigranulocytic or neutrophilic asthma. Compared with the control group, MS4A2, TPSAB1, FCER1A, TFF3, and TSPAN13 showed different levels of upregulation in both neutrophilic and paucigranulocytic samples. Among them, FCER1A showed the most significant upregulation in neutrophilic samples, while TFF3 showed the most significant upregulation in paucigranulocytic samples (Figures 5A,B). To elucidate the specific biological pathways associated with the five identified key genes, GSEA was performed (Figure 5C). The analysis revealed distinct pathway enrichments for each gene. The FCER1A gene was significantly enriched in pathways including Ribosome biogenesis, Herpes simplex virus 1 infection, Cornified envelope assembly, Epstein–Barr virus infection, and Cell cycle regulation. For the MS4A2 gene, enriched pathways comprised Protein processing within the endoplasmic reticulum, Cornified envelope formation, Olfactory signal transduction, Ribosomal function, and Mucin-type O-glycan biosynthesis. The TPSAB1 gene showed enrichment in pathways related to Endoplasmic reticulum protein processing, Mucin-type O-glycan biosynthesis, Herpes simplex virus 1 infection, Epstein–Barr virus infection, and Coronavirus disease (COVID-19). Enrichment for the TFF3 gene was observed in pathways such as Viral protein interactions with cytokine and cytokine receptors, Differentiation of Th1 and Th2 cells, Endoplasmic reticulum protein processing, Ribosomal function, and Herpes simplex virus type 1 infection. Finally, the TSPAN13 gene was enriched in pathways including Endoplasmic reticulum protein processing, Viral protein-cytokine receptor interactions, Protein export mechanisms, the nuclear factor kappa-light-chain-enhancer of activated B cells (NF-κB) signaling pathway, and N-glycan biosynthesis. Notably, all five key genes demonstrated enrichment in pathways associated with ribosomal function, endoplasmic reticulum protein processing, and immune-related factors. These findings establish a foundation for further exploration of the potential roles these key genes may play in immunomodulation and therapeutic strategies.

### 3.5 Receiver operating characteristic analysis of hub genes

**Figure 6:**
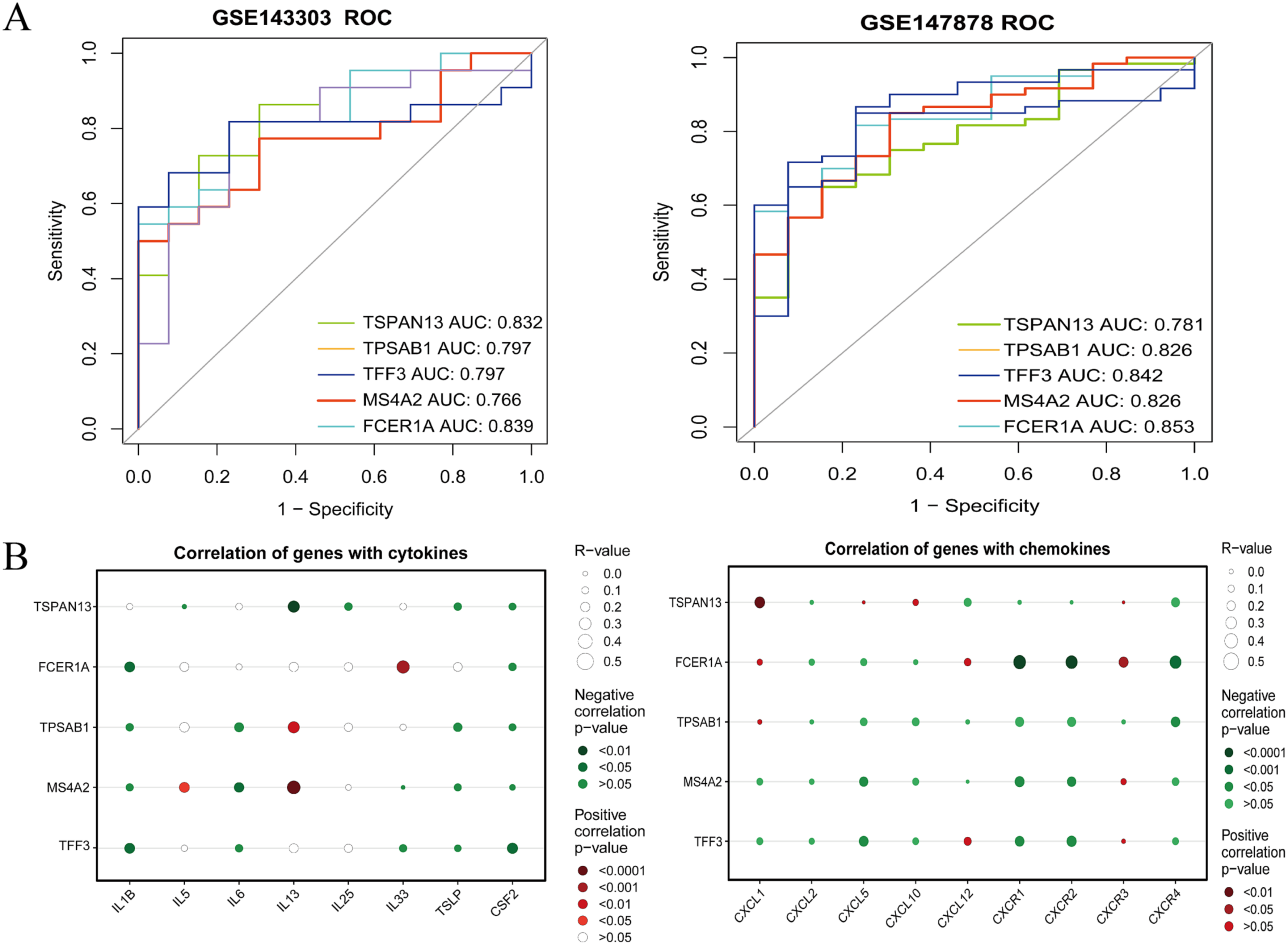
Identification of common genes related to ROC, chemokines, and cell receptors analysis. **(A)** ROC analysis of common genes. ROC curve for hub genes from the GSE143303 and GSE147878 were plotted with the pROC package in R. **(B)** Connection between core genes and chemokines and cell receptors.

The ROC curve was established using the external dataset GSE147878. As illustrated in Figure 6, ROC curve analysis was employed to evaluate the diagnostic potential of the five candidate key genes within asthma samples. The Area Under the Curve (AUC) values of the five hub genes, MS4A2, TPSAB1, FCER1A, TFF3, and TSPAN13, were all greater than 0.6, indicating their high predictive reliability. Integrating the gene expression profiles from both T2-high and non-T2 endotypes with the predictive performance of the model, MS4A2, TPSAB1, FCER1A, TFF3, and TSPAN13 were ultimately designated as common genes.

### 3.6 Relationship between hub DEGs and immune cells

**Figure 7:**
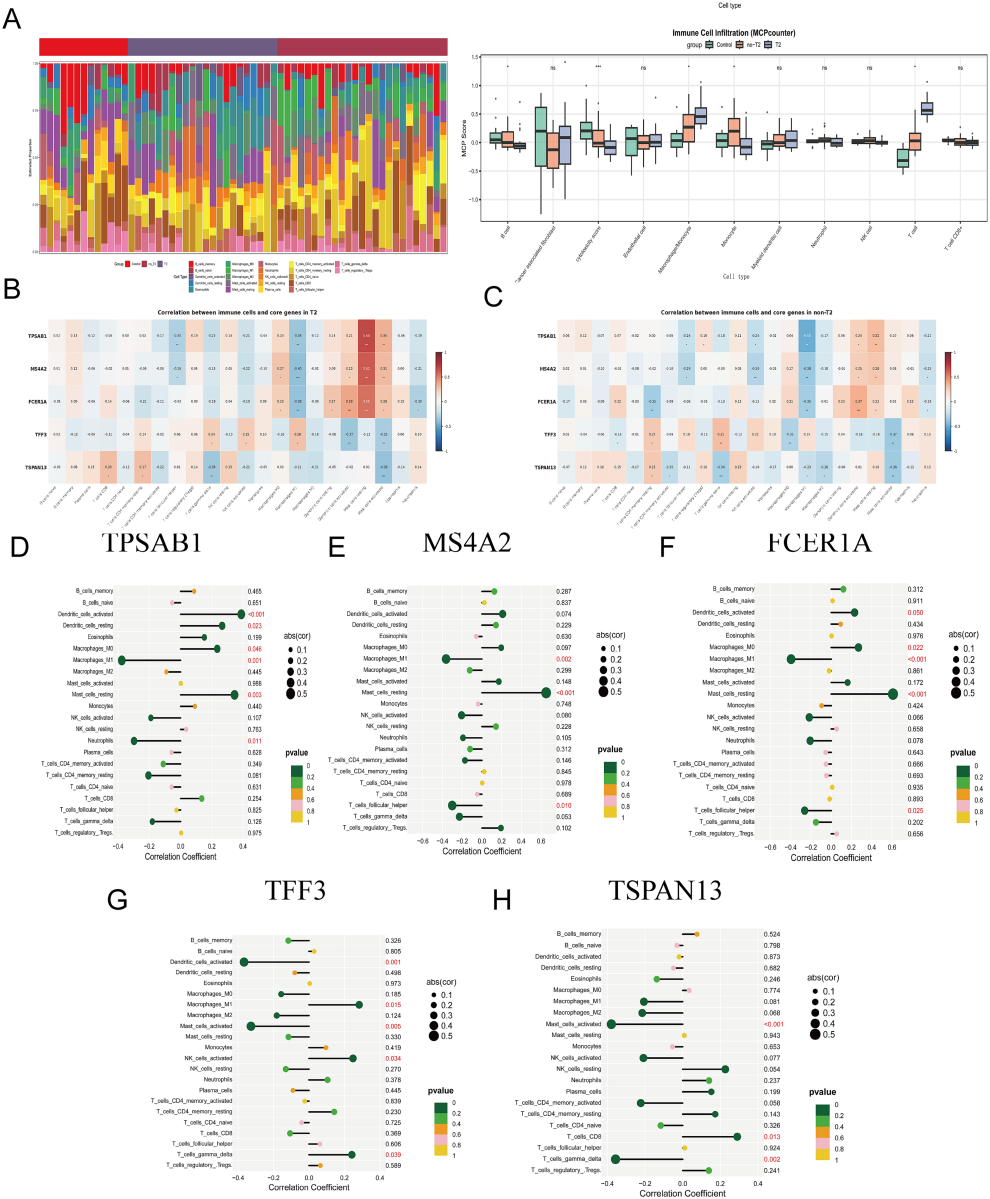
Correlation analysis of key genes and immune infiltration. **(A)** The proportion of 22 kinds of immune cells between the three groups. **(B)** The relationship between the common genes identified in the T2 group and immune cells.**(C)** The relationship between the common genes identified in the non-T2 group and immune cells. **(D–H)** The correlations between the expression of five hub genes (FCER1A, MS4A2, TSPAN13, TFF3, and TPSAB1) and immune cell enrichment.

It is well-established that immune responses and inflammatory processes are intricately linked to the pathogenesis of asthma. The proportions of infiltrating immune subpopulations were evaluated using CIBERSORT to examine the bronchial immune microenvironment in patients with T2 and non-T2 asthma. Figure 7A presents the infiltration ratios of different immune cells and the relationship between immune cells in bronchial samples of patients with T2-type asthma and those with non-T2-type asthma. Notably, the proportions of macrophages, and T cells in both the T2 and non-T2 groups were higher than those in the control group. Furthermore, in this study, we conducted a detailed analysis of the correlation between the expression levels of these 5 hub genes and various immune cells in the context of asthma (Figures 7B,C). This analysis unveiled strong associations, notably with resting mast cells in both T2 and non-T2 endotypes. In T2 and non-T2 group, MS4A2, TPSAB1, and FCER1A were remarkably relevant to resting mast cells. TFF3 correlated with T cells gamma delta, and TSPAN13 correlated with T cells CD4 memory resting. It should be noted that the CIBERSORT reference matrix was primarily developed from peripheral blood immune cell characteristics, and its application to airway tissues may introduce bias into estimates of resident cell populations.

Furthermore, the Spearman correlation coefficients between the expression levels of the five identified hub genes and various immune cell populations in the asthma context are presented in the lollipop graphs (Figures 7D–H). The analysis indicated that FCER1A and MS4A2 expression showed correlations with dendritic cells, macrophages, and mast cells. TPSAB1 expression was correlated with mast cells, while TFF3 expression demonstrated associations with T cells, NK cells, and mast cells. TSPAN13 expression was found to correlate with T cells. Additionally, the correlations between these five key genes and various immune-related factors, including chemokines and cellular receptors, were further elucidated (Figure 6B). These observed relationships underscore the significant involvement of immune cells in the pathogenic mechanisms of asthma. This investigation not only uncovers shared molecular mechanisms underlying immune infiltration in both T2 and non-T2 asthma but also provides a foundational experimental basis for developing therapeutic strategies that target the interactive network between immune cells and commonly dysregulated genes.

### 3.7 Gene-disease interaction network

Analysis of disease-disease associations can facilitate the study of pathological conditions and the identification of genomic commonalities across different diseases. The interrelationship between two disorders often stems from shared genetic components. Based on data from the DisGeNET database, the constructed gene-disease interaction network revealed associations for the five hub genes. The diseases demonstrating the strongest correlations with the studied hub genes were asthma, breast carcinoma, dermatitis, atopic hypersensitivity, and eczema (Figure 4C). Notably, the majority of these associated conditions are linked to immune dysregulation or inflammatory processes. These findings have significant implications for advancing the understanding of asthma mechanisms and for informing the development of related therapeutic approaches.

### 3.8 Single-cell atlas analysis of key gene expression

**Figure 8:**
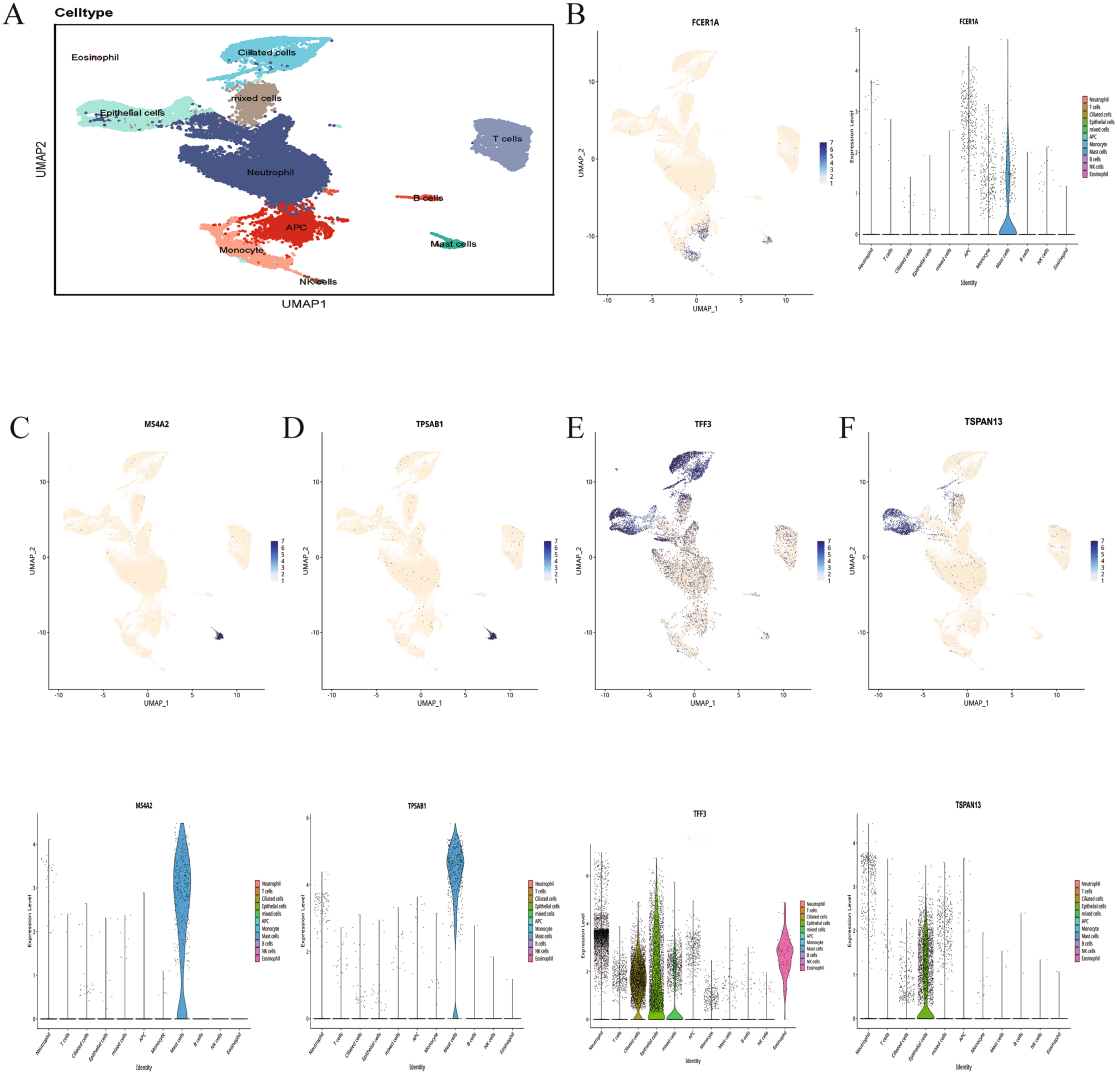
The expression patterns of key genes resolved at the single-cell level. **(A)** UMAP plot visualization displays 11 manually annotated cell clusters, providing a refined classification of the cell types. **(B–F)** Expression patterns of common genes at the single-cell level, visualized using a UMAP plot and a violin plot.

To determine the primary cellular origins and target cell types of five candidate genes, we analyzed single-cell RNA sequencing (scRNA-seq) data. After removing low-quality cells, unsupervised clustering was performed using UMAP, and cell clusters were annotated with the singleR package based on the human Primary Cell Atlas. Following manual curation, all clusters were further classified into eleven distinct cell types: T cells, B cells, eosinophils, epithelial cells, monocytes, antigen-presenting cells (APCs), mixed cells, neutrophils, natural killer (NK) cells, ciliated cells, and mast cells (Figure 8A).

Subsequently, UMAP projections and violin plots were employed to visualize the spatial distribution and expression levels of TPSAB1, MS4A2, FCER1A, TFF3, and TSPAN13 across these cell populations (Figures 8B–F). The analysis demonstrated that TFF3 expression was markedly elevated in ciliated and epithelial cells. In contrast, MS4A2, TPSAB1, and FCER1A showed significant upregulation in mast cells. TSPAN13 expression was notably increased primarily in epithelial cells.

### 3.9 Experimental validation of common gene expression in asthma

**Figure 9:**
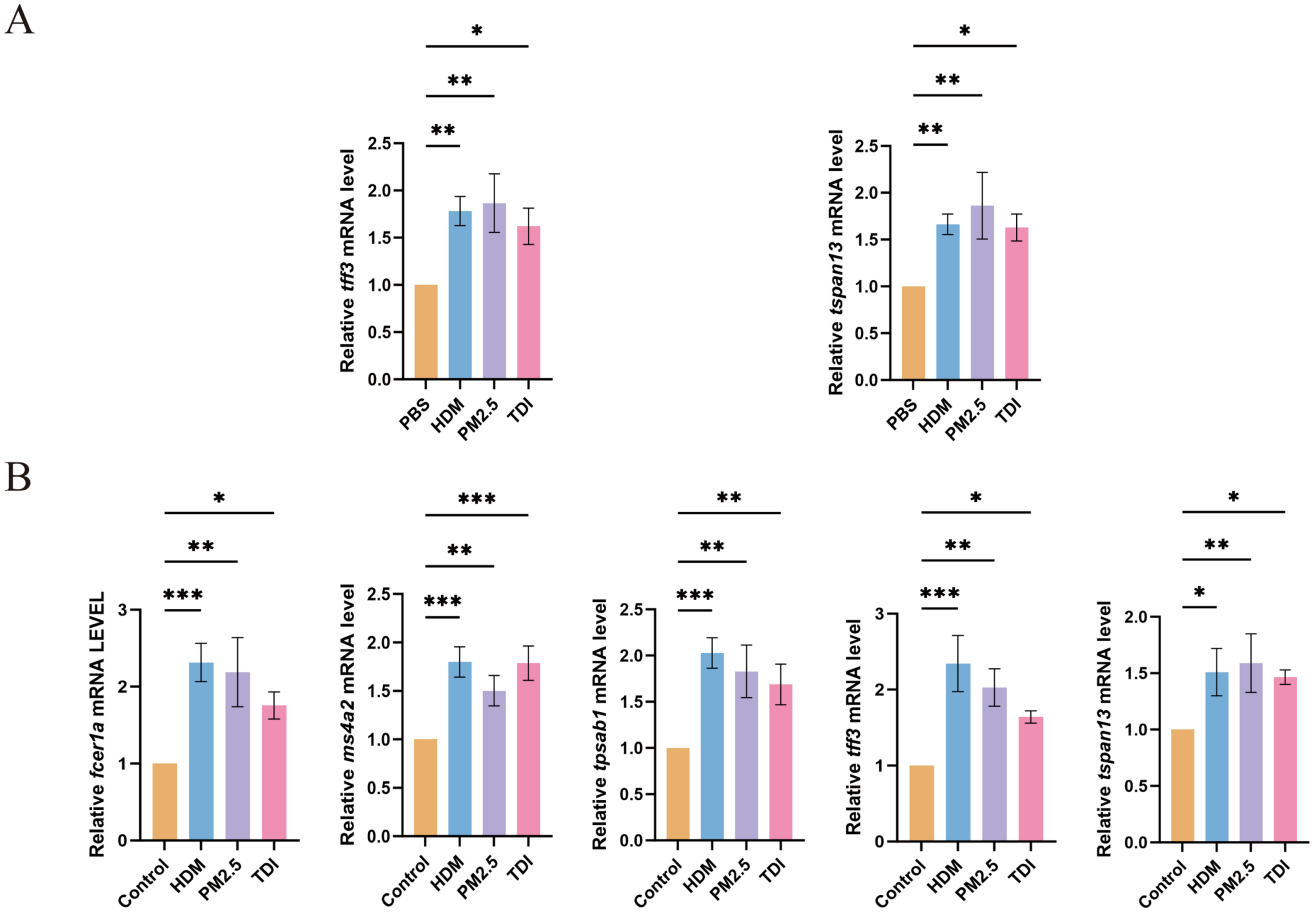
Verification of common genes in asthma model by qRT-PCR. **(A)** The transcriptional levels of *tff3* and *tspan13* in culture medium of HBE cells after treatment with PBS, HDM, PM2.5, and TDI for 24 h. **(B)** The transcriptional levels of *ms4a2, tpsab1, fcer1a, tff3* and *tspan13* in lung of mice. The data are represented as mean ± SD. * *p* < 0.05; ** *p* < 0.01; *** *p* < 0.001.

We next sought to preliminarily validate the involvement of these common genes in both T2 and non-T2 asthma through *in vitro* and *in vivo* experiments. A cellular model of T2 and non-T2 asthma was established by stimulating human bronchial epithelial (HBE) cells with house dust mite (HDM), PM2.5, and toluene diisocyanate (TDI). Quantitative real-time PCR (qRT-PCR) was used to measure mRNA levels of the common genes. Since MS4A2, FCER1A, and TPSAB1 are mainly expressed in mast cells rather than in airway epithelial cells. Therefore, no upregulation of these three genes was detected. Compared with the control group, expression of TFF3, and TSPAN13 was significantly increased in the HDM-, PM2.5-, and TDI-treated groups (Figure 9A).

**Figure 10:**
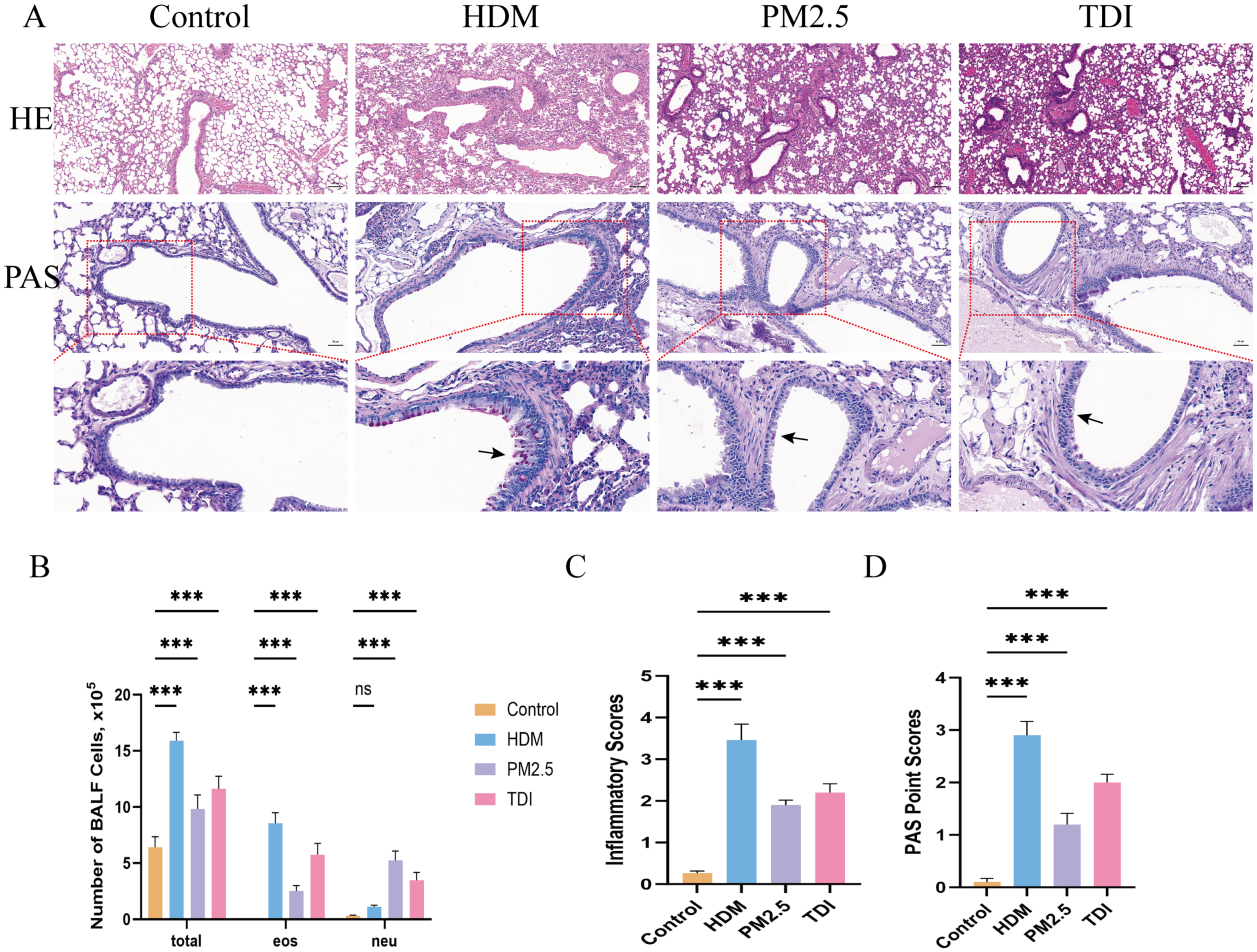
Establishment of asthma model in mouse. **(A)** H&E staining and PAS staining of representative lung sections. Black arrowheads indicate goblet cells containing mucus (magenta) after PAS staining. **(B)** Counts for eosinophils and neutrophils in BALF. **(C)** Inflammatory scores of lung sections from mice intranasally challenged with HDM, PM_2.5_, or TDI were calculated as described in Materials and methods. **(D)** PAS point scores of lung sections from mice intranasally challenged with HDM, PM_2.5_, or TDI were calculated as described in Materials and methods. *n* = 6 mice per group. BALF, bronchoalveolar lavage fluid. Total, total cells number in BALF; Eos, eosinophils; Neu, neutrophils. The data are represented as mean ± SD. ** *p* < 0.01; *** *p* < 0.001.

Three murine models of asthma-characterized by eosinophilic, neutrophilic, or mixed granulocytic airway inflammation-were generated by sensitization with HDM, PM2.5, or TDI, respectively. Hematoxylin and eosin (H&E) staining and inflammatory scoring of lung sections revealed pronounced inflammatory cell infiltration in the airways of HDM-, PM2.5-, and TDI-sensitized mice compared with controls (Figure 10A).

In HDM-challenged eosinophilic asthma models, total cell and eosinophil counts in bronchoalveolar lavage fluid (BALF) were significantly elevated, along with an increase in mucus-producing epithelial cells as shown by periodic acid-Schiff (PAS) staining. BALF from PM2.5-sensitized mice exhibited a marked rise in total inflammatory cells compared with HDM-, TDI-, and control groups, with neutrophils being the predominant cell type. TDI exposure induced a mixed infiltration of neutrophils and eosinophils in the airways (Figures 10B–D).

We verified the expression of the commonly identified genes from bioinformatics analyses using RT-qPCR (Figure 9B). The findings demonstrated a marked increase in the expression of these overlapping genes in asthma experimental models, aligning with the patterns observed in the transcriptomic datasets.

Collectively, our results suggest that MS4A2, TPSAB1, FCER1A, TFF3, and TSPAN13 play essential roles in the pathogenesis and progression of asthma.

## 4 Discussion

In this research, we retrieved the asthma-related datasets GSE143303, and GSE147878 from the Gene Expression Omnibus (GEO) database. Differentially expressed genes (DEGs) between T2 and non-T2 asthma were identified through cross-comparison of the two DEG lists, followed by Gene Ontology (GO) and Kyoto Encyclopedia of Genes and Genomes (KEGG) enrichment analyses. The results indicated that the DEGs were predominantly enriched in pathways associated with inflammation and immune responses, implying a possible inflammatory connection between T2 and non-T2 asthma subtypes. A protein–protein interaction (PPI) network was subsequently constructed, leading to the identification of five overlapping genes. Further in-depth enrichment analysis of these shared genes provided additional support for the initial hypothesis. To assess the potential of these genes as therapeutic or diagnostic biomarkers for distinguishing T2 from non-T2 asthma, we evaluated their diagnostic performance using receiver operating characteristic (ROC) curve analysis. Ultimately, five core genes-MS4A2, TPSAB1, FCER1A, TFF3, and TSPAN13-were pinpointed. These candidate biomarkers were further validated through reverse transcription quantitative polymerase chain reaction (RT-qPCR) analysis using biological samples in a wet laboratory setting.

FCER1A is expressed on basophils and mast cells (22). FCER1A encodes the alpha subunit of the high-affinity IgE receptor (FcεRI), a protein that plays a central role in immune responses (23). Among them, IgE plays a key role in the development of a T2 inflammatory response. When IgE binds to FcεRI receptors expressed on the surface of inflammatory dendritic cells, they work together to present allergens to memory Th2 lymphocytes, which in turn amplify antigen presentation (24). It is now believed that high expression of FCER1A is closely associated with various immune cell activations in the myocardial microenvironment, which is involved in the pathologic process of dilated cardiomyopathy (25). This also suggests a close relationship between FCER1A and immune responses, confirming its potential role in asthma.

The membrane-spanning 4-domain subfamily A member 2 (MS4A2), a component of the mast cell IgE receptor, is a key gene in the allergic cascade (26). A study suggests that the elevated interaction between MS4A2 and IgE in nasal polyps contributes to a poor postoperative prognosis in patients with eosinophilic chronic rhinosinusitis (27). MS4A2 mRNA abundance was elevated in eosinophilic asthma versus non-eosinophilic asthma patients (28). Similarly, in a case–control association study, the MS4A2 C-109 T T/T genotype was associated with asthma in the Chinese Han children (29). MS4A2 is believed to play a key role in atopy and asthma, as it can release cytokines and pro-inflammatory mediators that trigger inflammatory responses (30). Notably, the intracellular segment of MS4A2 contains an immunoreceptor tyrosine-based activation motif, which facilitates the formation of signal transduction complexes. Therefore, MS4A2 protein activation can regulate cytoskeletal remodeling, signal transduction cascades, transcriptional responses, and cellular differentiation. It is worth noting that Pan-cancer multi-omics profiling of MS4A2 has also been shown to be involved in colorectal cancer (CRC), demonstrating distinct activation features, and acting as a mast cell-driven gatekeeper of lung adenocarcinoma progression (31).

TPSAB1, an enzyme associated with mast cell regulation and function, plays a crucial role in host defense by modulating immune responses and inflammation (32). The current study demonstrated that the risk of severe anaphylaxis in humans is associated with inherited differences in *α*-tryptase-encoding copies at TPSAB1 (33). Meanwhile, TPSAB1 is involved in regulating airway inflammation in a mice model of asthma (34). Moreover, in patients with severe asthma, TPSAB1 transcripts are positively correlated with FENO, highlighting their role in the disease progression of asthma (28). In the pathological process of acute lung injury (ALI), tryptase released from mast cells mediates ALI induced by intestinal ischemia–reperfusion by activating PAR-2 to produce IL-8 (35). Notably, in Cutaneous melanoma patients, complement component C3 expression significantly correlates with the TPSAB1, and the high expression of both markers is linked with poorer melanoma survival (36).

Trefoil factor 3 (TFF3) is a 59-amino acid peptide that belongs to the trefoil factor family. Initially observed to be secreted by intestinal goblet cells and involved in the protection of the gastrointestinal tract against mucosal injury and subsequent repair (37). TFF3 mediates the NF-κB/Cyclooxygenase-2 (COX2)pathway to regulate the activation of polymorphonuclear myeloid-derived suppressor cells and attenuates experimental necrotizing enterocolitis in a T-cell-dependent manner (38). The current study showed increased secretion of TFF3 in the lungs of patients with chronic obstructive pulmonary disease (COPD), as well as significant increases in serum levels. This suggests a role for TFF3 in the pathogenesis of pulmonary diseases characterized by mucus hypersecretion (39).

Furthermore, the biological functions of TFF3 extend beyond mucosal defense. It is also involved in anti-apoptotic signaling, tumor cell migration and invasion, and the inhibition of anoikis in epithelial cells (39, 40). Airway inflammation and epithelial apoptosis are recognized as critical factors in the pathogenesis of asthma (41). Studies have shown that neutrophil apoptosis can mitigate TDI-induced airway hyperresponsiveness and inflammation. TFF3 modulates anti-apoptotic signaling by influencing inflammatory cell activation and mediator release, primarily through pathways such as phosphatidylinositol 3-kinase (PI3K)/protein kinase B (PKB/AKT) signaling (36). Collectively, current evidence indicates that TFF3 plays a significant role in the pathology of both T2 and non-T2 asthma, and its regulation of anti-apoptotic pathways may represent a shared mechanism underlying the development of these asthma endotypes.

TSPAN13 belongs to the transmembrane four superfamily, which is involved in mediating signal transduction processes essential for regulating cell migration, adhesion, activation, and apoptosis (40). In the context of human cancers, TSPAN13 is involved in the adhesion and signaling of immune cells, such as neutrophils and macrophages, contributing to the tumor cell proliferation, migration, and invasion process (41, 42). Studies have shown that TSPAN13 expression negatively correlated with CD8^+^ T cell infiltration in breast cancer patient samples. Mechanistically, TSPAN13 can enhance the ubiquitination of major histocompatibility complex class I (MHC-I) by recruiting STIP1 homology and U-Box containing protein 1 (STUB1), which in turn leads to immune evasion (43). Furthermore, another study confirmed that TSPAN13 enhances synergistic anti-inflammatory effects in macrophages. TSPAN13 plays a critical role in immune regulation and inflammation, which may be relevant to its involvement in asthma pathogenesis, although direct evidence is lacking.

The ribosome, an essential cellular complex, plays a critical role in cellular physiology by translating messenger RNAs into functional proteins (44). Disruption or impairment of ribosomal components can result in the synthesis of defective proteins. Epidemiological evidence indicates that ribosome-inactivating stress is linked to human mucosal epithelial disorders (45). In the present investigation, Gene Set Enrichment Analysis (GSEA) revealed that three hub genes-FCER1A, MS4A2, and TFF3-were significantly associated with ribosomal pathways, implying that these genes may modulate asthma progression through ribosomal mechanisms. Notably, Th1 and Th2 cell differentiation, endoplasmic reticulum protein processing, and viral infection pathways also exhibited substantial enrichment (Figure 5). These findings suggest that not only inflammatory responses but also diverse molecular pathways contribute to the pathogenesis of both T2 and non-T2 asthma. In conclusion, the pathophysiological mechanisms underlying T2 and non-T2 asthma are complex, involving environmental factors, genetic susceptibility, and immune dysregulation.

The immune pathways identified in this study underscore a considerable overlap between T2 and non-T2 asthma, indicating that immune system dysregulation may act as a shared underlying factor. Pathway analysis results demonstrate that the immune system occupies a central role in linking T2 and non-T2 asthma, with changes in immune cell profiles appearing particularly crucial in their interplay. Previous studies indicate that airway neutrophils are strongly associated with asthma severity and corticosteroid resistance (46). Neutrophilia is linked to enhanced T1 inflammation. Eosinophils, a predominant cell type in allergic asthma airways, are closely tied to T2 inflammation. However, interactions between neutrophils and eosinophils have been documented. Research by Wenzel et al. demonstrated that neutrophil levels in lung tissue were highest in the eosinophilic severe asthma subgroup (47). The innovative and clinically significant core finding of this study is that three classic mast cell markers (MS4A2, FCER1A, TPSAB1) are consistently upregulated in both type 2 and non-type 2 asthma, rather than being limited to type 2 inflammation. This result confirms that mast cell activation is a common pathological event in different asthma subtypes. Recent studies have shown significant functional heterogeneity among mast cells: IgE/FcεRI-activated mast cells are the primary effector cells in type 2 eosinophilic inflammation, while IL-33-stimulated mast cells can mediate neutrophilic inflammation in non-type 2 asthma (48). Furthermore, mast cells can mediate type 1/type 2 immune crosstalk by releasing cytokines such as TNF-*α*, CXCL10, and CCL5, promoting Th17-mediated neutrophil recruitment (49–51). This explains the elevation of mast cell signature genes in non-type 2 asthma. This shared characteristic of mast cell activation may represent a unified pathological mechanism for both type 2 and non-type 2 asthma, providing new targets for treating broad asthma phenotypes.

## 5 Limitation

There are some limitations in our research. First, the clinical data available in public databases is very limited. Since all three datasets are derived from bronchial biopsies or bronchial epithelial samples in comparable clinical settings, their intersection may not yield the broad validation implied by the term “common genes.” The reliability of the conclusion depends on the dataset’s heterogeneity and representativeness. If the dataset has sample bias or category imbalance, the model may deviate towards specific groups, potentially affecting the fairness of predictions. To enhance the model’s reliability, future research should use more diverse data to improve its stability. Second, our study is limited by data availability, and the conclusions are primarily based on transcriptomic data, which restricts the model’s generalizability to other scenarios. To enhance the model’s reliability, it is necessary to incorporate larger, multi-institutional datasets (e.g., foundational and clinical experimental studies) to derive more robust conclusions. Additionally, our analysis was conducted solely at the gene expression level, and the patient data obtained from public databases may include genetic factors, environmental influences, and geographic/racial differences. These elements were not considered in our analysis and may introduce selection bias, potentially limiting the universality of the study results. In the future, comprehensive research integrating environmental exposure data and genetic information will be crucial for improving the predictive capabilities of asthma models. Fourth, because suitable bioinformatics tools for analyzing immune cell infiltration in airway tissues were unavailable, we used the CIBERSORT algorithm to quantify immune cell types. It should be noted that the CIBERSORT reference matrix was primarily developed from peripheral blood immune cell characteristics, and its application to airway tissues may introduce bias into estimates of resident cell populations.

## 6 Conclusion

In summary, our integrated systems biology analysis revealed a common signaling pathway shared by T2 and non-T2 asthma endotypes. We identified five key genes-MS4A2, TPSAB1, FCER1A, TFF3, and TSPAN13-as shared biomarkers with potential as therapeutic targets. The immune pathways uncovered in this study demonstrate substantial overlap between T2 and non-T2 asthma, indicating that immune dysregulation may act as a unifying mechanism. These findings advance our understanding of asthma pathogenesis and provide a novel framework for future therapeutic development.

## Data Availability

The datasets presented in this study can be found in online repositories. The names of the repository/repositories and accession number(s) can be found in the article/Supplementary material.

## Footnotes

1
http://www.genemania.org
