# Supplementary material for: In silico identification and experimental validation of shared genes and exploration of molecular links between type 2 and non-type 2 asthma
Source: Front Med (Lausanne). 2026 May 8;13:1798063. doi: 10.3389/fmed.2026.1798063 (PMC13195886; doi:10.3389/fmed.2026.1798063)
Supplement: Supplementary file 1 [file Table_1.DOCX]

| **Table S1** The primers sequence for qRT-PCR | | |
| --- | --- | --- |
| Species | Primer name | Sequence（5'-3') |
| human | β-actin-F | GACTTAGTTGCGTTACACCCTTTCTTG |
| human | β-actin-R | ACTGCTGTCACCTTCACCGTTCC |
| human | TPSAB1-F | GTGACGCAAAATACCACCTTGGC |
| human | TPSAB1-R | CCATTCACCTTGCACACCAGGG |
| human | FCER1A -F | GTGGAGAATACAAATGTCAGCACC |
| human | FCER1A -R | CTCCATCACCACCTCAGCAGAG |
| human | MS4A2-F | AGACCAAGTGCTTTATGGCTTCC |
| human | MS4A2-R | GCTCCACAGATTGTGAGTGACAC |
| human | TFF3-F | TCCAGCTCTGCTGAGGAGTACG |
| human | TFF3-R | ATCCTGGAGTCAAAGCAGCAGC |
| human | TSPAN13-F | GATTTCCAGTCTCCGAGTGGT |
| human | TSPAN13-R | GGCTAAACAAGCGCAAGATACAG |
| mouse | β-actin-F | GGCTGTATTCCCCTCCATCG |
| mouse | β-actin-R | CCAGTTGGTAACAATGCCATGT |
| mouse | TPSAB1-F | GCCAATGACACCTACTGGATG |
| mouse | TPSAB1-R | GAGCTGTACTCTGACCTTGTTG |
| mouse | FCER1A -F | GAGTGCCACCGTTCAAGACA |
| mouse | FCER1A -R | GTAGATCACCTTGCGGACATTC |
| mouse | MS4A2-F | TGGTTGGTTTGATATGCCTTTGT |
| mouse | MS4A2-R | CACTGCACCCCAGAATGGATA |
| mouse | TFF3-F | TCCAAGCCAATGTATGGTGCCG |
| mouse | TFF3-R | CAGGGCACATTTGGGATACTGG |
| mouse | TSPAN13-F | CATCGGCTTTGGGCTGATCT |
| mouse | TSPAN13-R | TTCAGAGCTAAACAAGCACAAGA |
